# The Synthetic Genome Summer Course

**DOI:** 10.1093/synbio/ysy020

**Published:** 2018-11-27

**Authors:** Benjamin A Blount, Tom Ellis

**Affiliations:** 1 Imperial College Centre for Synthetic Biology, Imperial College London, London, UK; 2 Department of Bioengineering, Imperial College London, London, UK

**Keywords:** synthetic genome, SCRaMbLE, CRISPR, workshop, Sc2.0

## Abstract

The Synthetic Genome Summer Course was convened with the aim of teaching a wide range of researchers the theory and practical skills behind recent advances in synthetic biology and synthetic genome science, with a focus on Sc2.0, the synthetic yeast genome project. Through software workshops, tutorials and research talks from leading members of the field, the 30 attendees learnt about relevant principles and techniques that they were then able to implement first-hand in laboratory-based practical sessions. Participants SCRaMbLEd semi-synthetic yeast strains to diversify heterologous pathways, used automation to build combinatorial pathway libraries and used CRISPR to debug fitness defects caused by synthetic chromosome design changes. Societal implications of synthetic chromosomes were explored and industrial stakeholders discussed synthetic biology from a commercial standpoint. Over the 5 days, participants gained valuable insight and acquired skills to aid them in future synthetic genome research.

## 1. Introduction

Synthetic genome science was first highlighted to the wider scientific community by the announcement that a mycoplasma cell was functioning after having its genome replaced by an entirely chemically synthesized genome (1). The ability to design a genome *in silico*, albeit based predominantly on an existing genome sequence, and have that sequence function in a living cell opened up new possibilities for dissecting genome function and introducing design elements on a genome-wide scale. Subsequent projects have started to investigate this potential. Further iterations of the synthetic mycoplasma genome have focused on reducing the gene number to probe the genetic requirements of a minimal cell (2), whilst efforts to alter the codon usage of *Escheric*h*ia coli*, initially approached with top-down genome engineering approaches (3) have shifted to genome synthesis to capitalize on the volume of sequence alterations allowed by *in silico* design and chemical synthesis (4).

Perhaps the most ambitious project to exploit the potential of synthetic genome techniques is Sc2.0, the synthetic yeast genome project (5). The project aims to generate synthetic versions of all 16 *Saccharomyces cerevisiae* chromosomes, which deviate from the wild-type sequence following specific design criteria. Non-essential introns, tRNA genes and repetitive sequences have been removed from chromosome designs, with the aim of increasing stability. Extensive recoding has also taken place. As well as incorporating synthetic watermark sequences, PCRTags, into coding sequences, every TAG stop codon has been recoded to TAA for future TAG codon repurposing. This repurposing could potentially include encoding of non-natural amino acids for incorporation into peptides or alterations in codon usage for biocontainment or virus resistance (3, 6). To add even more functionality to the synthetic chromosomes, a symmetrical loxP recombination site has been inserted into the 3’ untranslated region (UTR) of every non-essential gene (7). And unlike in other projects, once Sc2.0 synthetic chromosomes are published, strains containing them become freely available for researchers and companies alike, with the hope that the strains will be a valuable resource for the wider scientific community (8). The design principles and technologies behind the Sc2.0 project are also likely to frame future synthetic genome projects, both in yeast and other organisms.

Yet the resources developed around synthetic genome projects, in terms of both approaches and strains, are being employed by relatively few research groups, despite their availability, ease-of-use and prominence in the scientific literature. To address this shortfall, we identified a summer course as a way to teach researchers from around the world the techniques involved in building a synthetic genome, with a focus on the design, assembly and implementation strategies of the Sc2.0 project. 

## 2. Educational goals

The Synthetic Genome Summer Course was devised as an opportunity for researchers to learn about synthetic genome, synthetic biology and genome engineering science. A key part of this was the opportunity to have practical techniques, and the theoretical background behind them, taught by the researchers who developed the techniques. Tutorials on relevant software tools and research talks from leading members of the field were also very important in supporting this. As well as acquiring practical skills and knowledge and increasing awareness of the developing technologies, we also wanted to encourage attendees to consider societal aspects of synthetic genomes and their possible implications. We also wanted to give researchers from diverse scientific backgrounds and career stages plenty of opportunity to network with others who shared an interest in synthetic genomes. Ideally, course attendees would learn first-hand from world-leading experts the theoretical background and practical skills required to engage in cutting-edge synthetic genome science.

**Figure 1.**
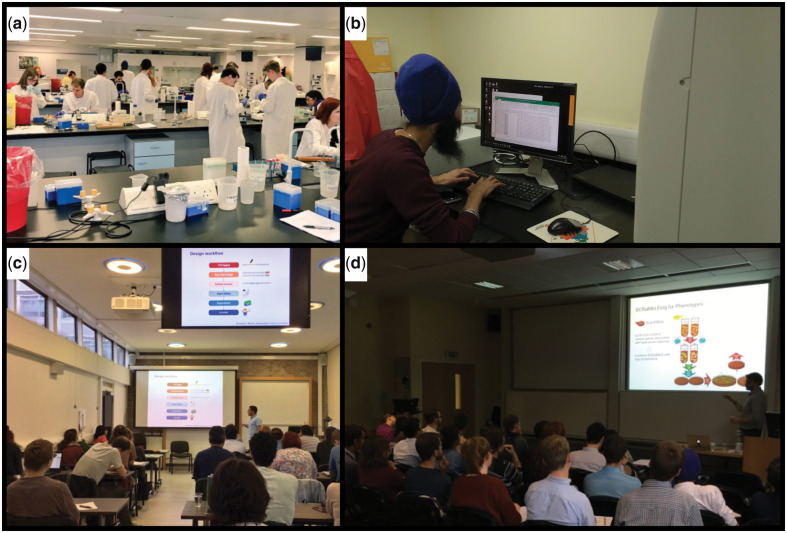
Photographs of the lab practicals (**a**), programming automated DNA assembly (**b**), the synthetic genome software tool workshop (**c**) and a SCRaMbLE tutorial (**d**).

The week-long course took place in Edinburgh, UK, on the 3–7 July 2016 and was attended by 30 people, selected from 64 applicants from around the world. Attendees had varied backgrounds covering biology, computer science, physics and social sciences from academia, industry and citizen science institutions (Table 1, Figure 1). Participants were based on 10 different countries and ranged in experience from undergraduate students to principle investigators.

**Table 1.** Participants in the Synthetic Genome Summer Course, July 2016

	Number of participants
Position	
Undergraduate students	2
Masters students	1
PhD students	12
Postdoctoral researchers	6
Research fellows	3
Principle investigators	1
Industry researchers	4
Citizen scientists	1
Country	
UK	18
China	2
France	2
Germany	2
Australia	1
Belgium	1
Netherlands	1
Singapore	1
Sweden	1
USA	1

Information on positions and countries in which participants are based is correct at the time of the course.

## 3. Taught aspects

The design, construction, debugging and exploitation of synthetic genomes involves a wide variety of skills and techniques. The taught theory and practical sessions of the workshop were designed to span a wide range of these aspects within the week-long timeframe. Table 2 lists the various tutorials, workshops, research talks and industry presentations that took place during the summer course.

**Table 2:** Taught aspects of the Synthetic Genome Summer Course, July 2016.

Title	Speaker
Tutorials	
Introduction to Sc2.0	Tom Ellis, Imperial College London, UK
Introduction to SCRaMbLE	Benjamin Blount, Imperial College London, UK
Introduction to Benchling	Hannah Shen, Benchling Inc., USA
Golden Gate Assembly	Benjamin Blount, Imperial College London, UK
Phenotype Debugging	Benjamin Blount, Imperial College London, UK
CRISPR	Will Shaw, Imperial College London, UK
Computer Aided Design and Analysis Methods for Synthetic Biology	Giovanni Stracquadanio, University of Essex, UK
Workshops	
SCRaMbLing the social: a safe space for strange questions	Jane Calvert and Erika Szymanski, University of Edinburgh, UK
Research Talks	
A standard workflow to assemble and phenotype synthetic yeast	Junbaio Dai, Tsinghua University, China
Genetics from scratch—designing and building synthetic chromosomes	Leslie Mitchell, New York University, USA
Tools for big DNA projects and a synthetic plastid genome	Jim Ajioka, University of Cambridge, UK
Programmable biological functionalities for autonomous microbial factories and therapeutics	Matthew Wook Chang, National University of Singapore
The propagation of perturbations in rewired gene networks	Mark Isalan, Imperial College London, UK
3C for metagenomic investigations	Romain Koszul, Institut Pasteur, France

Company	Speaker

Industry presentations	
Molecular devices	Dagmar Zunner
ThermoFisher	Andreas Stelzer
Labcyte	Carl Jarman
Autodesk	Florencio Mazzoldi
Merck	Michael Anderson-Burley
Gen9	Euan Forbes
Twist Bioscience	Emily Leproust

Information on affiliations is correct at the time of the course.

Tutorial sessions on the Sc2.0 project, Synthetic Chromosomal Recombination and Modification by LoxP-mediated Evolution (SCRaMbLE) (9), Clustered Regularly Interspaced Short Palindromic Repeats (CRISPR) mediated genome engineering (10), phenotype debugging and Golden Gate assembly (11) covered the theoretical background behind the techniques and the experiments performed during the practical sessions. A Tutorial on the Biostudio synthetic chromosome design software (7) was given by Giovanni Stracquadanio, demonstrating how sequences can be automatically refactored to incorporate specified design principles. Benchling virtual cloning and sequence management software was demonstrated by Hannah Shen, feeding into the use of Benchling to find CRISPR targets for use with the yeast CRISPR system covered by Will Shaw (12). More information on the content of the tutorials can be found in Supplementary Information 2.

A session to discuss the societal aspects of synthetic biology and synthetic genomes, run by Jane Calvert and Erika Szymanski, invited students to anonymously submit questions and opinions to be discussed by the group. The importance of transparency in financial beneficiaries, ensuring that societal benefits are distributed fairly and the confusion and ambiguity around some of the language used in the field were amongst the points of discussion.

Research talks were given by prominent researchers in the field. These talks demonstrated how synthetic genome engineering techniques—such as those taught in the Summer Course—are employed in cutting edge and high-impact international research. Additionally, talks were given by representatives from various companies involved in the field outlining the available technologies and products that can aid synthetic genome efforts.

## 4. Practical course

The practical sessions were split between three experimental workflows, devised to cover the various skills and techniques identified as being particularly important for synthetic genome science. Workflow 1 concentrated on the use of the SCRaMbLE system to generate diversity in semi-synthetic strains; Workflow 2 included CRISPR-based *in vivo* debugging of synthetic sequence, genomic preparation from colonies and polymerase chain reaction (PCR) screening for synthetic watermark sequences; and Workflow 3 covered the construction of heterologous pathways via automated combinatorial Golden Gate assembly and high efficiency transformation of DNA into yeast. The methods used can be found in the SGSC Handbook (Supplementary Information 1).

### 4.1 Practical workflow 1: SCRaMbLEing pathways and hosts

With the insertion of loxPsym recombination sites into the 3’ UTR of non-essential genes, synthetic chromosomes can undergo SCRaMbLE, whereby Cre recombinase activity is induced to cause insertions, deletions, duplications and inversions via recombination between unspecified loxPsym sites. This process can cause a huge amount of genetic diversity within a SCRaMbLEd population, leading to phenotypic variation (13–15).

To demonstrate the phenotypic diversity that can be generated by SCRaMbLE, both in a specific metabolic pathway and on a chromosomal scale, we offered the participants a choice of strains with which to perform their own SCRaMbLE experiments (Supplementary Information 1, pages 15–17, Table 3).

**Table 3.** Strains offered to participants for SCRaMbLE experiments

No	Strain	Strain notes	Plasmid^a^	Plasmid notes
1	synV × BY4742	Diploid strain generated by crossing synV (yXZX651) with BY4741—contains synV and WT chrV	pJCH017^b^	Violacein pathway without loxP sites
2	synV	Haploid yXZX651—contains synV	pJCH017^b^	Violacein pathway without loxP sites
3	synV	Haploid yXZX651—contains synV	pJCH052^b^	Violacein pathway flanked by loxPsym sites
4	synV	Haploid yXZX651—contains synV	pLM494^c^	β-Carotene pathway variant from pJC178 with all genes flanked by loxPsym sites
5	BY4742	Haploid strain—contains WT chrV	pLM494^c^	β-Carotene pathway variant from pJC178 with all genes flanked by loxPsym sites
6	synV	Haploid yXZX651—contains synV	pLM496^c^	β-Carotene pathway variant from pJC181 with all genes flanked by loxPsym sites
7	BY4742	Haploid strain—contains WT chrV	pLM496^c^	β-Carotene pathway variant from pJC181 with all genes flanked by loxPsym sites
8	synXI.A-L	Haploid strain—contains partially constructed synXI		

^a^All strains also contained pSCW11-creEBD.

^b^pJCH017 is described in Blount *et al.* (15), pJCH052 has the same violacein pathway but with loxPsym sites 3 bp downstream of the stop codon of every CDS.

^c^Pathways in pLM494 and pLM496 are described in Mitchell *et al.* (16), as pJC178 and 175 respectively and were subsequently assembled into pLM292 and pLM496 with a pRS406-derived backbone.

In groups of two, participants were given the freedom to devise and perform their own experiments with any of the strains but were also offered some suggested experiments. The most popular suggested experiment was to SCRaMbLE strains with either the native BY4742 or a synthetic chromosome V (16) background, each with a plasmid-encoded loxPsym-formatted beta-carotene pathway. Two different pathways were available for this experiment, with different promoters driving the pathway genes. Before SCRaMbLEing, the first pathway variant (pLM494, derived from pJC178) leads to a yellow colony color due to a higher proportion of the yellow/orange beta-carotene product compared to the red lycopene intermediate, whilst the second pathway variant (pLM496, derived from pJC181) leads to a pink-orange colony color as proportionately more lycopene is produced (17). By SCRaMbLEing just the pathway, in the case of BY4742 background strains, or the pathway and a synthetic chromosome, the diversity of pathway output generated in these two contexts can be compared.

**Figure 2.**
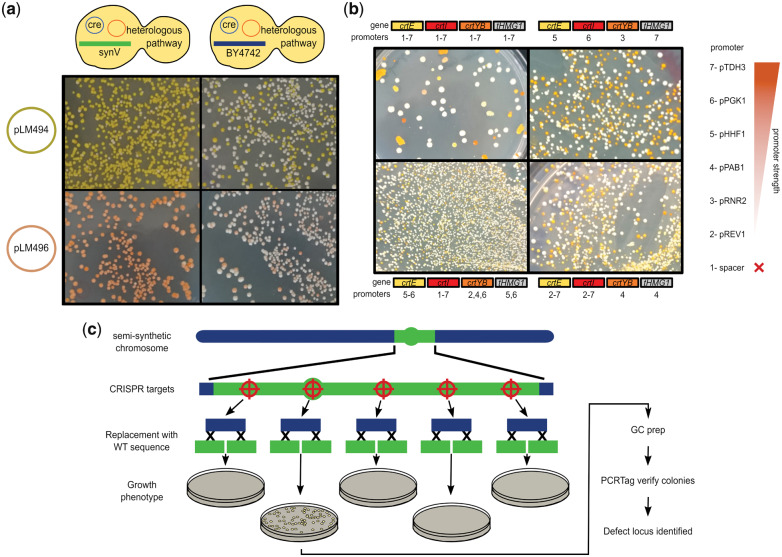
Lab-based practical work. (**a**) A typical example of colonies on plates following SCRaMbLE induction for 4 h. The genetic background of the strain is indicated at the top of the photographs and the beta-carotene pathway plasmid to the left. (**b**) Examples of transformation plates following transformation of beta-carotene pathway libraries assembled by Golden Gate to the teams’ specification. Further examples are available online (18). The promoter ranges chosen to drive expression of each pathway gene are indicated either above or below each photograph. To the right, the promoters corresponding to each promoter strength number are indicated. (**c**) A diagrammatical representation of the CRISPR-based defect debugging process performed by participants.

SCRaMbLE was induced in exponential phase cultures with beta-estradiol for 4 h, prior to washing, plating and incubating for 2 days. SCRaMbLE of the pathways in a BY4742 background led to a larger proportion of white colonies than in the synV background, indicating that some, or all, of the pathway genes had been lost in these colonies (Figure 2a). In the colonies that retained the pathways, the synV background seemed to produce a wider range of color intensity, representing pathway output. This is particularly true with plasmid pLM496. It was speculated that in a synV background, the larger number of available loxP sites results in fewer recombination events that delete a crucial beta-carotene pathway component. The wider range of expression levels indirectly observed in a synV background may be due to chromosomal recombination events affecting pathway expression or could simply be a result of more colonies retaining a functional pathway giving a larger sampling space.

As well as gaining first-hand experience SCRaMbLEing synthetic chromosomes and pathways, effects of chromosomal and pathway context on the SCRaMbLE process were demonstrated.

### 4.2 Practical workflow 2: automated combinatorial pathway construction

Expression of heterologous pathways is a common aim of synthetic biology projects and recent advances in combinatorial DNA assembly and automated liquid handling have drastically improved the throughput of pathway variant testing. Groups were asked to design combinatorial libraries of beta-carotene pathways by specifying what promoters were to drive expression from four coding sequences, *crtE*, *crtI*, *crtYB* and *tHMG1.* For each position, groups were given an option of inserting six different promoters or a non-functional spacer sequence. For each gene, a single fixed promoter or a defined combination of promoters could be specified depending on whether the aim was to generate colonies accumulating a particular pathway product or to produce as much diversity in pathway outputs as possible. Varying levels and proportions of the orange beta-carotene and pathway intermediates, such as the red pigment lycopene, give colonies different colors (17). This gives an easy visual representation of pathway output variation for the purposes of this exercise. Once each of the 15 groups had decided upon their promoter combinations, they submitted them to the demonstrators and Golden Gate reactions using the YTK format were assembled by an Echo acoustic liquid handler (Labcyte), and the assembly reactions were performed using standard conditions in a thermocycler (19). The completed reaction mixes were transformed directly into BY4741 using a high efficiency lithium acetate heat shock method (20). Transformants were then plated on selective media and incubated for 2 days (Supplementary Information 1, pages 18–19).

Each team picked different promoter variables for their beta-carotene pathway library, resulting in noticeable differences in the variety of colony color on the transformation plate. Invariably, around half of the colonies were white, indicating an incorrect assembly (Figure 2b). In less time-restricted workflows, this could be avoided by adding a linear DNA-specific nuclease step or transforming into an *E. coli* intermediate propagation strain. Nevertheless, transforming assembly reactions directly into the yeast host strain consistently resulted in many successful transformants. Variety in colony color and, by implication, levels of the different pathway products, increased with the number of potential promoters at each position (Figure 2b). Teams with fixed promoters at every position saw high consistency in colony color, whilst those who specified the largest ranges of promoter strength saw the widest variety of colony color, ranging from pale yellow to orange, pink and red.

As well as using automation to build combinatorial plasmid libraries and high efficiency transformation techniques to generate large numbers of transformants, participants demonstrated that altering the range of expression levels sampled in the pathway library constrained or relaxed the phenotypic space generated.

### 4.3 Practical workflow 3: CRISPR-mediated *in vivo* debugging of synthetic sequence

A major challenge of synthetic chromosome design and construction is the difficulty in identifying the causes of unexpected phenotypic defects introduced by synthetic sequence. If a strain suffers reduced fitness following the replacement of native chromosomal DNA with a redesigned synthetic equivalent, it is likely that the cause of the defect lies in a design change made to that section of synthetic DNA. As the defect is an unintentional result of one of a potentially large number of changes, a systematic way of narrowing the search space is needed. One way to achieve this is to use a CRISPR system to generate double-stranded breaks at various loci throughout the suspect sequence and replace sections of synthetic DNA with the native sequence. Transformants with revertant phenotypes can be screened for wild-type DNA insertion, indicating that the corresponding synthetic locus is contributing to the defect.

To demonstrate this, we supplied teams with strain synXI.M, a BY4742 strain in which ∼40 kb of chromosome XI has been replaced during a single round of integration by synthetic sequence redesigned to Sc2.0 specification, but leading to a slow growth phenotype. Teams were asked to select one of five loci at which substantial design changes have been implemented in the synthetic sequence that could feasibly be causing the defect: locus 1 was the RPL12A gene, which has its intron removed; locus 2 was the CEN11 centromeric sequence, which was now flanked by loxPsym sites; locus 3 was YKR005C, which has its intron removed; locus 4 was a site at which a tRNA had been removed; and locus 5 was the YPT52 coding sequence, in which two sections have been recoded to incorporate PCRTag watermarks. For each locus, teams were supplied with a DNA mix containing a two-part CRISPR system (12) encoding Cas9, a yeast selectable marker and a gRNA targeting Cas9 to a synthetic PCRTag sequence at the locus, and a linear section of corresponding wild-type DNA to repair the double strand break and eliminate the CRISPR target site (Supplementary Information 1, pages 18–20, Figure 2c).

Teams transformed the CRISPR DNA mixes into synXI.M using the high efficiency lithium acetate heat shock method, plated onto selective media and incubated for 2 days. The growth defect in synXI.M prevented visible colony formation within 2 days and so any visible colonies represented transformants in which the defect had been fixed. Genomic DNA was isolated from colonies using the GC prep method (21). PCR with primers targeting either the recoded synthetic PCRTag watermarks or the corresponding wild-type sequences were used to verify the replacement of synthetic sequence with the wild-type template. Using this workflow, a team successfully identified a colony fixed for the growth defect that was verified by PCRTag analysis to have had the CEN11 centromeric region reverted to the wild-type sequence, confirming that design changes in this region were the source of the growth defect.

## 5. Discussion

The Synthetic Genome Summer Course was conceived as a way to educate a broad range of researchers on the skills, methodologies and theoretical background behind recent advances in synthetic biology and synthetic genome science, leading to more widespread uptake. Through tutorial sessions, software workshops, research talks and industry presentations, attendees learnt about these approaches. Over the three practical workflows, participants gained hands-on experience with many relevant experimental techniques including SCRaMbLE, CRISPR, Golden Gate assembly, high efficiency DNA transformation, phenotype debugging and synthetic chromosome watermark analysis.

Anonymous feedback was received from 21 of the participants and was extremely positive, with responders rating the overall course a mean average 4.6 out of 5 and particularly highlighting the breadth of techniques learnt, the relevance of the course content and the networking opportunities offered. Almost all responders believed that they would use specific techniques and skills acquired during the course in their future research and several participants have subsequently authored research articles making use of the techniques taught (15, 22, 23). The full responses to feedback questions are collated in Supplementary Information 3 —Anonymous Feedback Responses.

Previous summer schools and workshops, such as the International Synthetic and Systems Biology Summer School (24) have featured sessions discussing synthetic genomes, along with wider synthetic and systems biology themes. The Synthetic Genome Summer Course, however, was unique in its focus on synthetic genome science and in giving students the opportunity to have hands-on experience working with synthetic genome strains whilst learning from leaders in the emerging field. Given the excellent feedback from participants, and the continuing developments in synthetic genome science, this course can hopefully be built upon to deliver future courses and educate more researchers in the latest ideas and techniques in the field.

## Supplementary Material

Supplementary Information 1

Supplementary Information 2

Supplementary Information 3
