# Supplementary material for: The Synthetic Genome Summer Course
Source: Synth Biol (Oxf). 2018 Nov 27;3(1):ysy020. doi: 10.1093/synbio/ysy020 (PMC7445779; doi:10.1093/synbio/ysy020)
Supplement: Supplementary Information 1 [file ysy020_supplementary_information_1.pdf]

Course Handbook

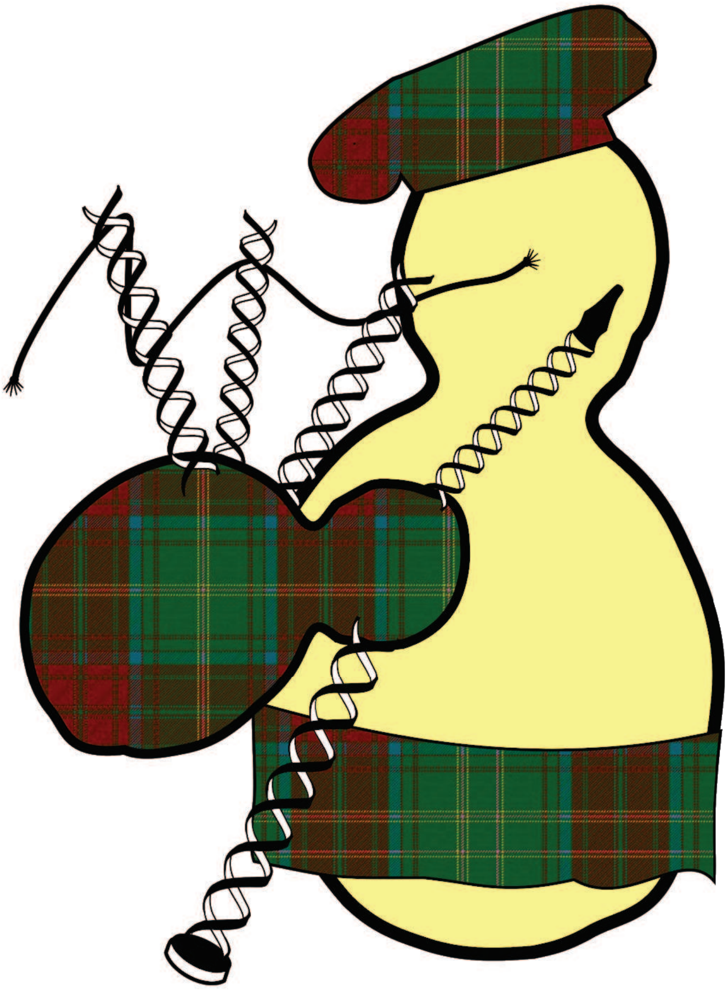

Synthetic Genomes
Summer Course
July 2016 Edinburgh

Name:

Introduction

Welcome to the first Synthetic Genomes Summer School! In this booklet you'll find all the information you need both for the workshop plus some handy tips for your spare time.

Please make sure that you keep this booklet with you every day, as it contains all of the protocols for the practical work, along with a timetable.

We hope you enjoy the course and the city of Edinburgh. See you in the first session!

Internet access

The University of Edinburgh campus broadcasts the Eduroam network. If your institution is part of Eduroam, you can access the network by entering your login details as you would for your own institution's network.

Alternatively, we have also arranged guest accounts that you can use.

Contents

Introduction	2
Internet access	2
Contents	2
Workshop organisers	3
Participants	5
Programme	12
Protocols	15
Experiment 1 - SCRaMbLE	15
Experiment 2 - CRISPR	18
Maps & bus routes	21
Contact information	24
Notes	25
Sponsors	27

Workshop Organisers

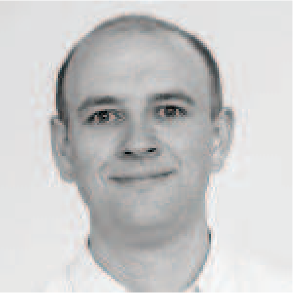

Tom Ellis
I'm Tom, born and educated in the UK with a spell doing research in the USA too. I run the Ellis Lab at Imperial College London overseeing many cool projects, including the construction of the yeast synthetic chromosome XI, engineering bacterial cellulose and investigating the effects of synthetic networks on host cells. I try to spend my days coming up with exciting new ideas and answering way too many emails.

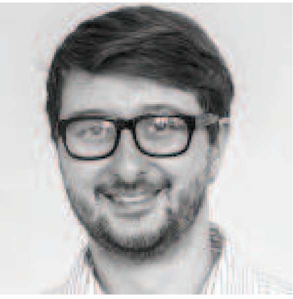

Ben Blount
I'm Ben, a postdoc in the Ellis Lab at Imperial College London, where I'm heading the construction of synthetic yeast chromosome XI as part of the Sc2.0 project. I also work with DNA binding proteins that can be retargeted to specific DNA sequences to affect gene expression. Outside of the lab I like brewing beer and watching bands that play songs in pretentious time signatures.

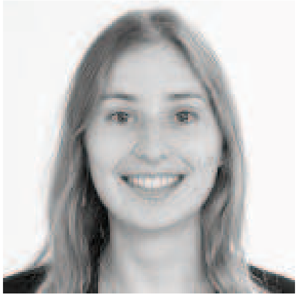

Maureen Driessen
Hi, I‘m Maureen, a yeast fan from the Netherlands. I'm working on the construction of synthetic yeast chromosome XI in the Ellis Lab as part of the Sc2.0 project. In my spare time I often get away from London to go hiking.

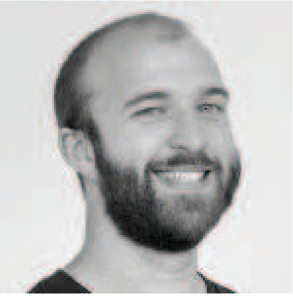

Rob McKiernan
Hey I'm Rob, otherwise known as the "Computer Monkey" of the Ellis Lab. I take care of the programming side of the Sc2.0 project at Imperial College London, as well as coding any other bits and pieces for others. When I'm not working I like to travel, play saxophone and simulate near death experiences through boarding sports.

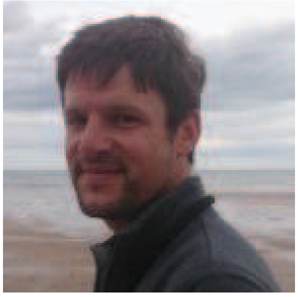

Alistair Elfick
Alistair Elfick is Professor of Synthetic Biological Engineering and Depute Director of the UK Centre for Mammalian Synthetic Biology at the University of Edinburgh. He co-author the influential book Synthetic Aesthetics: Investigating Synthetic Biology’s Designs on Nature (MIT Press). Researching at the boundary of life and the inorganic, Alistair seeks to enable humankind to more sympathetically harness biology’s ability to sustainably manufacture.

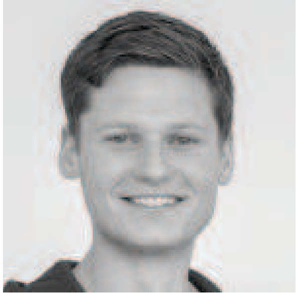

Will Shaw
I’m Will, a PhD student in the Ellis Lab at Imperial College London. My main research focuses on refactoring yeast signalling pathways for repurposing as biosensors, but I’m interested in anything yeast synbio. When I’m not working I like fishing, playing squash and going to the pub.

Programme

PH = Pollock Halls of Residence
DR-LT = Daniel Rutherford Building Lecture Theatre
DR-Lab = Daniel Rutherford Building Teaching Laboratory
Cai = Patrick Cai Group Laboratory
EGF = Edinburgh Genome Foundry

Sunday 3 July

14:00	Welcome and introductions	PH
15:30	Introduction to Sc2.0	DR-LT
16:00	Introduction to SCRaMbLE	DR-LT
17:00	Introduction to Benchling - Hannah Shen	DR-LT

Monday 4 July

09:00	Experiment 1 - SCRaMbLE	DR-Lab
09:30	Discussion of experiment 1 - SCRaMbLE	DR-LT
10:00	Coffee	
10:30	Industry speaker - Molecular Devices - Dagmar Zunner	DR-LT
10:50	Guest Speaker - Junbaio Dai - *"A standard workflow to assemble and phenotype synthetic yeast"*	DR-LT
11:40	Industry speaker - ThermoFisher - Andreas Stelzer	DR-LT
12:00	Lunch	
13:00	Experiment 1 - SCRaMbLE	DR-Lab
13:30	Introduction to experiment 3 - Golden Gate Assembly	DR-LT
14:00	Industry Speaker and demo - Labcyte - Carl Jarman	DR-LT
14:30	Introduction to experiment 3 - Echo	DR-LT
15:00	Coffee	
15:30	Experiment 3 - Golden Gate Assembly	Cai
17:00	Experiment 1 - SCRaMbLE	DR-Lab

Tuesday 5 July

9:00	Jane Calvert and Erika Szymanski - *“SCRaMbLing the social: a safe space for strange questions”*	DR-LT
11:00	Coffee	
11:30	Official team photo	
11:40	Introduction to phenotype debugging	DR-LT
12:30	Lunch	
13:30	Introduction to experiment 2 - CRISPR	DR-LT
14:30	Experiments 2 & 3, CRISPR/Golden Gate Assembly	DR-Lab

Wednesday 6 July

09:00	*In silico* resources	DR-LT
10:00	Coffee	
10:30	Guest Speaker - Giovanni Stracquadanio - *“Computer Aided Design and Analysis Methods for Synthetic Genomics”*	DR-LT
11:30	Industry Speaker - Autodesk - Florencio Mazzoldi	DR-LT
11:50	Guest Speaker - Michael Anderson-Burley - *“How Synthetic Biology is being used at Merck”*	DR-LT
12:30	Lunch	
13:30	Guest Speaker - Leslie Mitchell - *"Genetics from scratch - designing and building synthetic chromosomes"*	DR-LT
14:15	Guest Speaker - Jim Ajioka - *“Tools for big DNA projects and a synthetic plastid genome*”	DR-LT
15:00	Coffee	
15:30	Industry Speaker - Gen9 - Euan Forbes	DR-LT
15:50	Industry Speaker - Twist - Emily Leproust CEO	DR-LT
16:20	Experiment 1 - SCRaMbLE	DR-Lab
16:50	Discussion of experiment 2 - CRISPR	DR-LT

Thursday 7 July

09:00	Experiment 2 - CRISPR	DR-Lab
10:30	Coffee	
11:00	Guest speaker - Matthew Chang - “*Programmable biological functionalities for autonomous microbial factories and therapeutics”*	DR-LT
11:45	Guest Speaker - Mark Isalan - *"The propagation of perturbations in rewired gene networks"*	DR-LT
12:30	Lunch	
13:30	Guest Speaker - Romain Koszul - *“3C for metagenomic investigations”*	DR-LT
14:15	SynBioBeta short talks	EGF
15:00	Experiments 1, 2 & 3 - SCRaMbLE, CRISPR & Golden Gate Assembly	DR-Lab
16:30	Discussion of results	DR-Lab

Friday 8 July & Saturday 9 July

The 5th Annual Sc2.0 & Synthetic Genomes Conference in Edinburgh

---

Protocols

Experiment 1 - SCRaMbLEing pathways

There are several strains available for SCRaMbLEing

1. synV diploid with plasmid-borne violacein pathway.
2. synV haploid with plasmid-borne violacein pathway.
3. synV haploid with plasmid-borne violacein pathway, pathway is flanked by loxPsym sites.
4. synV haploid with plasmid-borne β-carotene pathway, each CDS with downstream loxPsym sites. Promoter variant 1.
5. BY4742 with plasmid-borne β-carotene pathway, each CDS with downstream loxPsym sites. Promoter variant 1.
6. synV haploid with plasmid-borne β-carotene pathway, each CDS with downstream loxPsym sites. Promoter variant 2.
7. BY4742 with plasmid-borne β-carotene pathway, each CDS with downstream loxPsym sites. Promoter variant 2.
8. synXI.A-L haploid.

Suggested experiments:

Experiment 1A - SCRaMbLE strains 1, 2 and 3. Which causes the most variation in violacein production?

Experiment 1B - SCRaMBLE strains 4 and 5 or 6 and 7. What is the difference between the semi-synthetic and non-synthetic genetic backgrounds?

Experiment 1C - SCRaMbLE strain 8 and incubate plates at 37°C. Are the SCRaMbLEd cells different to the negative control?

Day One - Monday 4th July

9:00 - 9:30 Inoculating cultures for SCRaMbLEing
You will be provided with culture tubes containing 5 ml of SDO HIS^- URA^- (for strains 1-7) or SDO LEU^- (for strain 8) media. To each, add 50 µl of overnight culture of the strains you want to grow up using a flame to maintain sterility. For each strain, inoculate 2 cultures, one to be SCRaMbLEd and one to act as a negative control. Remember to label your tubes with your initials and strain number! Place tubes in the rack to be incubated shaking at 30 °C.

13:00 - 13:30 SCRaMbLE induction
Be very careful when handing β-estradiol, it's toxic. When finished handling, dispose of gloves and put on a new pair
For each strain you should have 2 grow-up cultures. Under a flame, add 2 µl 5mM β-estradiol to one of the tubes per strain, marking clearly on the tubes which one has been induced with β-estradiol and which has not.

Place tubes in the rack to be incubated shaking at 30 °C.

17:00-18:30 Washing and plating
Label 1 fresh 1.5 ml microtube per culture with your initials, the strain number and whether or not that culture has been induced (e.g. I or U). Transfer 1 ml of each culture into the corresponding microtube. Spin down tubes for 10 minutes at 8000 rpm in a microfuge. Invert tubes ONCE over a liquid waste pot to remove supernatant (at this point, a cell pellet might not be visible). Add 1 ml DPBS to each tube and mix gently by pipetting up and down.

The strains will need to be diluted in DPBS prior to plating:

Strain number	Induced dilution to plate	Uninduced dilution to plate
1	1/100	1/100
2	1/10	1/100
3	1/10	1/100
4	1/10	1/100
5	1/100	1/100
6	1/10	1/100
7	1/100	1/100
8	1/10	1/100

Dilute each washed culture to the correct levels in a fresh 1.5 ml microtube and plate 100 µl onto the appropriate selective media agar plates under a flame. For strains 1-7, plate onto SDO URA- agar. For strain 8, plate onto YPD agar.

Day Three - Wednesday 6th July

16:30-17:00 Inoculating overnight cultures
Check plates for pickable colonies and inoculate 5 ml broths with any colonies of interest. If colonies are not yet pickable, this will not be a problem.

Day Four - Thursday 7th July

15:00-16:30 Results
Check your plates and see what’s happened.

Experiment 2 - Using CRISPR to debug synthetic genomes

Day Two - Tuesday 5th July

14:00-17:30 Transforming CRISPR DNA into yeast

The M megachunk of synXI causes a severe growth defect when integrated. CRISPR will be used to replace synthetic DNA regions with wild-type sequence to isolate which chunk (or chunks) is causing the problem.

Decide which chunk you want to revert to wild-type with CRISPR:

Chunk	Description
M1syn	the RPL12A intron has been removed
M2syn	loxPsym sites now flank the centromere
M3syn	the YKR005C intron has been removed
M4syn	a tRNA has been removed
M5syn	YPT52 PCRTag codons changed

Whilst performing the CRISPR transformation, you will be also given a vial of plasmid DNA assembled by Golden Gate Assembly using the Echo in experiment 3 to transform into BY4742.

1. Spin down the 2 tubes containing synXI.M culture and the 2 tubes containing BY4742 at 1000 g for 10 minutes.
2. Pour off the supernatant into a waste pot and resuspend each pellet in 10 ml **0.1 M** LiOAc. Pool the vials of synXI.M together into 1 tube and, separately, pool the vials of BY4742 together into 1 tube. Spin down the 2 tubes at 1000g for 10 minutes.
3. Pour off the supernatant into a waste pot and resuspend each pellet in 200 µl **0.1 M** LiOAc. Transfer 2 100 µl aliquots of each culture into fresh labelled 1.5 ml microtubes (4 tubes in total).
4. To each tube add 10 µl boiled ssDNA (vortex ssDNA before adding). After adding the ssDNA, mix briefly by vortexing on a medium setting.
5. Add 10 µl of the appropriate transformation DNA to each tube (10 µl dH_2O for one each of the BY4742 and synXI.M tubes, CRISPR DNA for the other synXI.M tube and Golden Gate Assembly DNA for the other BY4742 tube). Mix briefly by vortexing on a medium setting.
6. Incubate at room temperature for 30 minutes

7. Whilst tubes are incubating, prepare the transformation mix in a 15 ml Falcon tube. Add reagents in the order shown and mix by inversion:

Volume	Reagent
3 ml	50% PEG
450 µl	**1 M** LiOAc
500 µl	DMSO
450 µl	dH_2O

8. Once the 30 minutes is over, add 880 µl of the transformation mix to each tube, mix by pipetting.
9. Incubate at room temperature for 30 minutes.
10. Incubate at 42 °C in the heated block for exactly 14 minutes, mix halfway through by inversion.
11. Spin down for 2 minutes at 8000 rpm in a microfuge.
12. Carefully remove the supernatant with a pipette and resuspend each pellet in 250 µl 5 mM $CaCl_2$. Incubate at room temperature for exactly 10 minutes (start timing after resuspending the first pellet, then continue with the other tubes).
13. Once the first cell suspension has been incubated for 10 minutes, plate it all on one plate and then move on to the next cell suspension, and so on. The transformants should be plated on SDO URA^- media.

Day Four - Thursday 7th July

9:00-11:00 PCRTag verifying CRISPR colonies

Perform GC preps on CRISPR transformant colonies as follows:

1. Pick 3 colonies of interest from the CRISPR transformation plate and resuspend each in one of the 1.5ml tubes containing 5% chelex-100 and glass beads.
2. Vortex the tubes on the highest setting for 4 minutes.
3. Incubate the tubes at 100 °C in the heated block for 2 minutes.
4. Remove the tubes from the heated block. Be careful, they will be hot.
5. Spin tubes in a microfuge at top speed for 1 minute. While the samples are spinning, label a fresh microtube for each sample.

6. Transfer 10 µl from the very top of each sample into the corresponding fresh tube. Be careful not to transfer across any of the beads, using a 20 µl tip will help with this. The transferred supernatant is the gDNA prep.

To perform PCRTag PCRs on the gDNA, you will be assembling 2 reactions per gDNA sample, 1 to amplify from a wild-type PCRTag pair and 1 to amplify from a synthetic PCRTag pair. You will be given the appropriate primers for the CRISPR experiment you performed.

Set up 2 master mixes, 1 for each primer pair:

Volume in Master Mix (5x)	Volume per reaction	Reagent
-	0.2 µl	gDNA
25 µl	5 µl	GoTaq Green 2X Master Mix
5 µl	1 µl	Forward Primer
5 µl	1 µl	Reverse Primer
14 µl	2.8 µl	dH_2O

For each master mix, transfer 9.8 µl into 3 labelled PCR tubes. For easy viewing of the gel later, a good order for the PCR tubes is:

Tube 1	Tube 2	Tube 3	Tube 4	Tube 5	Tube 6
WT primers gDNA 1	Syn primers gDNA 1	WT primers gDNA 2	Syn primers gDNA 2	WT primers gDNA 3	Syn primers gDNA 3

To each tube, add 0.2 µl of the appropriate gDNA sample.

Once the PCR reactions are assembled, pass them to a demonstrator.

Notes

We thank our sponsors
