# Supplementary material for: The Synthetic Genome Summer Course
Source: Synth Biol (Oxf). 2018 Nov 27;3(1):ysy020. doi: 10.1093/synbio/ysy020 (PMC7445779; doi:10.1093/synbio/ysy020)
Supplement: Supplementary Information 2 [file ysy020_supplementary_information_2.docx]

**Supplementary Information 2 - Tutorial Session Summaries**

**Introduction to Sc2.0**

An overview of the Sc2.0 project, including:

- The history of the project and other synthetic genome projects
- The aims of the project
- Design changes implemented in the synthetic genome
- Genome assembly strategy and techniques
- Future benefits of the project and applications for the strains

Participants learnt about synthetic genome projects and, particularly, the principles behind the Sc2.0 project. This establishes the foundational knowledge required to understand the context of further tutorial and practical sessions.

**Introduction to SCRaMbLE**

An explanation of the SCRaMbLE system, including:

- How the cre-loxP system causes chromosomal rearrangements
- How the beta-estradiol induction system works
- Previous studies on SCRaMbLE-generated sequence diversity
- The practical workflow for inducing SCRaMbLE
- Previous results in using SCRaMbLE to optimise pathway expression
- An overview of practical workflow 1: SCRaMbLEing pathways and hosts
- A discussion of what phenotypes might be targeted for improvement with SCRaMbLE

Participants learnt about the theory behind the inducible SCRaMbLE system and the design criteria engineered into the Sc2.0 strains to allow this novel function. Participants also learnt the methods used in practical workflow 1, and the factors influencing their choice of experiment variant were discussed.

**Introduction to Benchling**

An interactive demonstration on how to use the web-based Benchling software, including:

- How to import custom DNA sequences or import from an existing database
- How to organise sequences into projects, check modification history and maintain version control
- How to annotate sequences, both automatically and manually
- How to perform virtual cloning and virtual PCR
- How to align sequences
- How to find CRISPR target sites

Participants learnt how to use sequence management and virtual cloning software. Of particular relevance to the practical workflows was the ability to perform virtual Golden Gate Assembly (practical workflow 2) and how to find CRISPR targets (practical workflow 3, CRISPR tutorial).

**Golden Gate Assembly**

An explanation of Golden Gate Assembly and practical workflow 2, including:

- The theory behind Golden Gate Assembly
- Applications of Golden Gate Assembly
- Automating Golden Gate Assembly
- The beta-carotene pathway genes
- The available promoters for combinatorial assembly of a pathway
- Potential results that teams could choose to aim for in their libraries
- Reaction conditions
- Pathway component selection

Participants learnt about Golden Gate Assembly in a general sense and also with specific relevance to the automated beta-carotene pathway assembly of practical workflow 2. Options for library specifications were discussed and each team of two decided on their promoter selections for their library.

**Phenotype Debugging**

An introduction to approaches to debugging synthetic sequences that are not functioning as specified, including:

- What are fitness defects?
- How to identify fitness defects using varied media conditions
- Common causes of fitness defects in the Sc2.0 project
- Introduction to the fitness defect to be debugged in practical workflow 3
- Possible causes of this defect
- Alternative debugging strategies, including *in silico* sequence analysis, analysing megachunk transformant pools and backcrossing
- Introduction to CRISPR
- Gap repair strategies to increase CRISPR efficiency
- Using CRISPR to debug a synthetic sequence
- Available CRISPR mixes for debugging in practical workflow 3
- Protocol for high efficiency yeast DNA transformations
- GC preps for fast and reliable DNA isolation from colonies
- Theory behind PCRTag analysis
- PCR reaction conditions

Participants learnt how to identify and troubleshoot fitness defects introduced by synthetic sequences. The protocols and theory behind practical workflow 3 were explained and options for CRISPR mixes were discussed. The high efficiency DNA transformation protocol to be employed in practical workflows 2 and 3 was explained, as well as the transformant colony screening process.

**CRISPR**

This session gave a more in-depth interactive tutorial on how to implement CRISPR in yeast, including:

- The plasmids making up the Ellis Lab CRISPR system
- Selecting a CRISPR target using Benchling
- Retargeting the gRNA component using custom oligonucleotides
- Designing repair template sequences
- Multiplexing CRISPR to target multiple sites
- Marker cycling for fast iterative CRISPR rounds

Participants learnt how the components of the CRISPR mixes in practical workflow 3 were designed and generated and also learnt how to implement CRISPR more generally in yeast for various applications.

**Computer Aided Design and Analysis Methods for Synthetic Biology**

An introduction to the design and analysis of a synthetic genome sequence using the software tools developed during the Sc2.0 project, including:

- Selecting design criteria
- Automated refactoring of a chromosome sequence to incorporate criteria with Biostudio
- Recoding restriction sites for downstream assembly
- Incorporation of PCRTag watermarks
- Analysing sequence changes
- Browsing synthetic sequences
- Generating component oligonucleotides/chunks for DNA synthesis

Participants learnt how the synthetic chromosomes that they used in the practical sessions were designed and how alternative synthetic chromosomal sequences with different design criteria can be generated.
