# Supplementary material for: The Synthetic Genome Summer Course
Source: Synth Biol (Oxf). 2018 Nov 27;3(1):ysy020. doi: 10.1093/synbio/ysy020 (PMC7445779; doi:10.1093/synbio/ysy020)
Supplement: Supplementary Information 3 [file ysy020_supplementary_information_3.docx]

**Supplementary Information 3 - Anonymous Feedback Responses**

Scales for answers:

Q3: 1-bad. 3-OK. 5-great

Q5: 1-too much lab work. 3-perfect balance. 5-too many talks

Q1: How would you describe your current position?

- Academic - Postdoc/Fellow/PI

Q2: Did you receive a bursary and/or travel award?

- Yes

Q3: How would you rate the Synthetic Genome Summer Course overall?

- 5

Q4: Are you likely to find what you learned on the Synthetic Genome Summer Course useful for your future research or industrial work, and if so how?

Most important part was meeting people from both academy and industry.

Q5: How would you rate the balance between lab practical classes and talks?

- 2

Q6: Is there anything you would like to have learned on the course that was not included?

many people missed to do the whole process (From design to the lab results) for the experiments we did. The design part (i.e. gRNA, GG part design, etc) could have been explained in more detail. The benchling tutorial was too fast to learn anything.

Q7: How did you find the social activities on the course?

Great. Maybe just missing some touristic visit to the city centre

Q8: If you were in charge next year, how would you change the course?

I would have included short/flash talks of the participants, to know better the kind of research each of us is doing

Q9: Would you recommend the course to someone else? Why/Why Not?

sure. Nice talks, great speakers, interesting people, good science, amazing dinners, etc.

Q10: In one or two sentences, summarise what you would tell our funders about your experience on the course.

The course was a important step in our career development where we had the opportunity to meet interesting people and learn a lot from them. We also had some hand on experiences that allow us to bring the knowledge back to our labs.

Q1: How would you describe your current position?

- Undergraduate/Other

Q2: Did you receive a bursary and/or travel award?

- Yes

Q3: How would you rate the Synthetic Genome Summer Course overall?

- 5

Q4: Are you likely to find what you learned on the Synthetic Genome Summer Course useful for your future research or industrial work, and if so how?

yes, as SCRaMbLE and CRISPR are likely to be used in my research during my postgraduate or phD projects

Q5: How would you rate the balance between lab practical classes and talks?

- 4

Q6: Is there anything you would like to have learned on the course that was not included?

A very brief reminder of transformations, cloning and general cloning (VERY BRIEF!!) just to remind anybody who has not done it in a while. Anything that was relevant and/or new that you would not come across otherwise...like SCRaMbLE.

Q7: How did you find the social activities on the course?

AMAZING!!!....I would introduce a "Rest day" to allow for city viewing and general rest.

Q8: If you were in charge next year, how would you change the course?

See above.

Q9: Would you recommend the course to someone else? Why/Why Not?

Definitely!!! The networking opportunities, especially for an undergraduate were amazing. I now have friends! Also an amazing learning opportunity of new techniques.

Q10: In one or two sentences, summarise what you would tell our funders about your experience on the course.

As an undergraduate, this has been one of the most amazing and informative courses I have been on. I met people that I can work with and learnt emerging techniques not found on my undergraduate course. This was an amazing experience and coupled with amazing classmates it was the best thing I've done during my undergrad journey.

Q1: How would you describe your current position?

- Postgraduate - Masters or PhD student

Q2: Did you receive a bursary and/or travel award?

- No

Q3: How would you rate the Synthetic Genome Summer Course overall?

- 5

Q4: Are you likely to find what you learned on the Synthetic Genome Summer Course useful for your future research or industrial work, and if so how?

Definitely. The GC preps and CRISPR experiments should be useful for a really broad range of yeast work, so I can see myself using them a lot. The SCRaMbLE experiments are obviously more specific but were nevertheless incredibly valuable. These were particularly useful to me as I plan to do some SCRaMbLE as part of my PhD project.

Q5: How would you rate the balance between lab practical classes and talks?

- 4

Q6: Is there anything you would like to have learned on the course that was not included?

Yeast mating, sporulation, tetrad analysis etc.

Q7: How did you find the social activities on the course?

Great!

Q8: If you were in charge next year, how would you change the course?

More lab work

Q9: Would you recommend the course to someone else? Why/Why Not?

Yes! Great introduction to Sc2.0 and for developing basic skills for working with yeast. Also get to meet plenty of people.

Q10: In one or two sentences, summarise what you would tell our funders about your experience on the course.

Fantastic course that provided a great overview of yeast genetic engineering and the Sc2.0 project. I learned some really valuable skills that I can now apply to my PhD project.

Q1: How would you describe your current position?

- Postgraduate - Masters or PhD student

Q2: Did you receive a bursary and/or travel award?

- No

Q3: How would you rate the Synthetic Genome Summer Course overall?

- 4

Q4: Are you likely to find what you learned on the Synthetic Genome Summer Course useful for your future research or industrial work, and if so how?

The experience of how to use SCRaMbLE and Crispr is very useful for my future research. I am the first year PhD student and I'll study the yeast in the near future. So my future research will be basded on this.

Q5: How would you rate the balance between lab practical classes and talks?

- 4

Q6: Is there anything you would like to have learned on the course that was not included?

About how to use Gibson assembly.

Q7: How did you find the social activities on the course?

It is very nice.

Q8: If you were in charge next year, how would you change the course?

I want to add more social activities.

Q9: Would you recommend the course to someone else? Why/Why Not?

Yeah, it is a wonderful experience.

Q10: In one or two sentences, summarise what you would tell our funders about your experience on the course.

I knew many useful skills to gene editing and got some new friends. This is a wonderful experience.

Q1: How would you describe your current position?

- Undergraduate/Other

Q2: Did you receive a bursary and/or travel award?

- No

Q3: How would you rate the Synthetic Genome Summer Course overall?

- 3

Q4: Are you likely to find what you learned on the Synthetic Genome Summer Course useful for your future research or industrial work, and if so how?

I know some new reaserch directions that I did not know before. And I also learnt some new technology about CRISPR and Hi-3C. I think it can broaden our eyes about synthetic biology.

Q5: How would you rate the balance between lab practical classes and talks?

- 3

Q6: Is there anything you would like to have learned on the course that was not included?

Will taught the way to design plasmid our gRNA or others. It is so quickly that I can't know well. I want the course can give more time about this.

Q7: How did you find the social activities on the course?

I think it is great. Social activities make me have more chance to communicate with them and know more.

Q8: If you were in charge next year, how would you change the course?

Respondent skipped this question

Q9: Would you recommend the course to someone else? Why/Why Not?

Yes, I would like to recommed the course to someone. I think this is a good chance to communicate with many different research fields people.

Q10: In one or two sentences, summarise what you would tell our funders about your experience on the course.

I make many friends in this course and learn many different thoughts of different research fields. It is a good experence.

Q1: How would you describe your current position?

- Academic - Postdoc/Fellow/PI

Q2: Did you receive a bursary and/or travel award?

- No

Q3: How would you rate the Synthetic Genome Summer Course overall?

- 3

Q4: Are you likely to find what you learned on the Synthetic Genome Summer Course useful for your future research or industrial work, and if so how?

Respondent skipped this question

Q5: How would you rate the balance between lab practical classes and talks?

- 1

Q6: Is there anything you would like to have learned on the course that was not included?

I would have liked to spend much more time with the software design tools, both becoming familiar with the interfaces but also understanding additional details about practical design consideration.

Q7: How did you find the social activities on the course?

Pretty good but poorly paced. We had a lot of awkward time between items on the schedule. I would rather have had items move faster and/or be more tightly paced and then had a solid block of time to take a rest or go for a hike.

Q8: If you were in charge next year, how would you change the course?

I get that a synbio course seems that it should have lab work, but the only thing the lab work added to my experience was bonding time with my table. Everything we heard in the week devalued the lab work, nearly all of the actual experimental design had been done for us in advance, and yet we spent a lot of time centrifuging things when we could have been learning design skills.

Q9: Would you recommend the course to someone else? Why/Why Not?

I don't know. On the one hand, I saw an interesting breadth of what the field is currently doing from both academic and industry sides. On the other, I didn't learn a lot about doing synthetic biology, and I was pretty put off by the number of talks we had that were really just advertisements for specific product lines.

Q10: In one or two sentences, summarise what you would tell our funders about your experience on the course.

A great chance to meet diverse and universally interesting colleagues in synthetic biology, but I would have loved to spend more hands-on time learning practical design skills.

Q1: How would you describe your current position?

- Academic - Postdoc/Fellow/PI

Q2: Did you receive a bursary and/or travel award?

- No

Q3: How would you rate the Synthetic Genome Summer Course overall?

- 5

Q4: Are you likely to find what you learned on the Synthetic Genome Summer Course useful for your future research or industrial work, and if so how?

Yes definitely, we have already been talking about using CRISPR in our work.

Q5: How would you rate the balance between lab practical classes and talks?

- 3

Q6: Is there anything you would like to have learned on the course that was not included?

Personally I would have liked a little more detail on building plasmids etc for CRISPR and golden gate.

Q7: How did you find the social activities on the course?

Really good, meals were all nice and social activities were fun. Could have done with some "Shots on Rob" though.

Q8: If you were in charge next year, how would you change the course?

Spread out the sponsor talks over the day. Rather than in a block together. No role play session.

Q9: Would you recommend the course to someone else? Why/Why Not?

Yes definitely. Excellent course well run and very interesting

Q10: In one or two sentences, summarise what you would tell our funders about your experience on the course.

Excellent course with a good mix of lab and theoretical work. Smoothly run by enthusiastic scientists.

Q1: How would you describe your current position?

- Academic - Postdoc/Fellow/PI

Q2: Did you receive a bursary and/or travel award?

- No

Q3: How would you rate the Synthetic Genome Summer Course overall?

- 4

Q4: Are you likely to find what you learned on the Synthetic Genome Summer Course useful for your future research or industrial work, and if so how?

Yes, the high efficiency transformation protocol looks to be an improvement on my current method.

Q5: How would you rate the balance between lab practical classes and talks?

- 4

Q6: Is there anything you would like to have learned on the course that was not included?

I would have liked to have learnt more about the CRISPR method, and in particular the design aspects of it, eg what promoter was used to drive the guide RNA and Cas9 etc.

Q7: How did you find the social activities on the course?

Very good.

Q8: If you were in charge next year, how would you change the course?

I appreciate that they have sponsored the event but I felt there were too many talks by companies promoting their (very expensive!) machinery.

Q9: Would you recommend the course to someone else? Why/Why Not?

Yes, but probably to someone that had limited experience working with yeast.

Q10: In one or two sentences, summarise what you would tell our funders about your experience on the course.

It was a very informative course that provided an interesting insight into the future of yeast synthetic biology.

Q1: How would you describe your current position?

- Postgraduate - Masters or PhD student

Q2: Did you receive a bursary and/or travel award?

- Yes

Q3: How would you rate the Synthetic Genome Summer Course overall?

- 5

Q4: Are you likely to find what you learned on the Synthetic Genome Summer Course useful for your future research or industrial work, and if so how?

Yes, I'm considering a move to work with yeast and/or keen to try out using yeast for DNA assembly. I also feel the greater knowledge of SCRaMbLE as a tool for directed evolution and CRISPR as a 'debugging' tool will be invaluable in future projects. I already knew about Benchling but I still learned about new features which will likely be very useful. I also think the industry speakers really helped give a feel of the sort of work different companies do which is really useful for career planning.

Q5: How would you rate the balance between lab practical classes and talks?

- 4

Q6: Is there anything you would like to have learned on the course that was not included?

I'm really keen to try the multipart 'in yeasto' assembly that was mentioned a couple of times (once in Leslie Mitchell's talk I think?), so it would be cool if this could be featured.

Q7: How did you find the social activities on the course?

Really great variety of food and activities, the atmosphere was great all week!

Q8: If you were in charge next year, how would you change the course?

If there is any way that participants could be given more choice and freedom about what genetic circuits they SCRaMbLE that would be cool, or alternatively, a variety of selection pressures could be offered e.g. temperature and caffeine were discussed at Sc2.0. I think it would be cool if the colony growth and colour/intensity could be quantified within the workshop, there is tonnes of information generated that was not analysed.

Q9: Would you recommend the course to someone else? Why/Why Not?

Absolutely, had fun and learned loads.

Q10: In one or two sentences, summarise what you would tell our funders about your experience on the course.

This course is one of a kind in its content and also applies to such a broad range of scientists it really should be supported as much as possible. The venue was excellent and tieing in with the Sc2.0 conference worked really well. Synthetic Biology is growing rapidly but I think few scientists really know what it is about, so we need more opportunities to get scientists trained in the mindset of applying engineering principles to biology. The organisers and instructors did a tremendous job.

Q1: How would you describe your current position?

- Industry Researcher

Q2: Did you receive a bursary and/or travel award?

- No

Q3: How would you rate the Synthetic Genome Summer Course overall?

- 5

Q4: Are you likely to find what you learned on the Synthetic Genome Summer Course useful for your future research or industrial work, and if so how?

Yes, some of the tools covered iduring the course were very interesting, including the pathways scrambling and crispr

Q5: How would you rate the balance between lab practical classes and talks?

- 3

Q6: Is there anything you would like to have learned on the course that was not included?

Although some detils on the CRISPR technology were not covered directly, ti was possible to have a chat with people doing the work and get advice from them

Q7: How did you find the social activities on the course?

Very well organised and distributed.

Q8: If you were in charge next year, how would you change the course?

Could we have a bit more lab work?

Q9: Would you recommend the course to someone else? Why/Why Not?

The course provided a very good summary of some of the current technologies used in yeast molecular biology. It was really interesting to catch up with some of the most recent projects and activities led by world leading researchers

Q10: In one or two sentences, summarise what you would tell our funders about your experience on the course.

It was an extremely positive experience to have joined the Summer School as it covered a range of topics currently used in yeast synthetic biology. There as a well

Q1: How would you describe your current position?

- Academic - Postdoc/Fellow/PI

Q2: Did you receive a bursary and/or travel award?

- Yes

Q3: How would you rate the Synthetic Genome Summer Course overall?

- 3

Q4: Are you likely to find what you learned on the Synthetic Genome Summer Course useful for your future research or industrial work, and if so how?

Yes, have switched the lab cloning to golden gate and the MoClo kit already.

Q5: How would you rate the balance between lab practical classes and talks?

- 5

Q6: Is there anything you would like to have learned on the course that was not included?

Would have liked a more detailed session on CRISPR and the actual design of the plasmids.

Q7: How did you find the social activities on the course?

Brilliant!

Q8: If you were in charge next year, how would you change the course?

Less industry talks - the big sell seemed wasted on some of the audience that have little to no say in lab budgets. NO MERCK! Some pre-attending prep work for benchling to get everyone familiar with it and perhaps some papers on topics that would be covered so that in general the attendees would all be on similar footing. More lab time and break away sessions on specific topics people want covered on the last day.

Q9: Would you recommend the course to someone else? Why/Why Not?

Yes but to early MSc/PhD students with little lab experience

Q10: In one or two sentences, summarise what you would tell our funders about your experience on the course.

Excellently organised event, covering many hot topics in SynBio and molecular biology. Well attended by a broad cross section of academic and industry scientists.

Q1: How would you describe your current position?

- Undergraduate/Other

Q2: Did you receive a bursary and/or travel award?

- No

Q3: How would you rate the Synthetic Genome Summer Course overall?

- 5

Q4: Are you likely to find what you learned on the Synthetic Genome Summer Course useful for your future research or industrial work, and if so how?

Yes. My lab has been preparing to collaborate with NYU on chromosome 9, so the info about Sc2.0 was very helpful. The CRISPR tricks I learned will also prove useful to us.

Q5: How would you rate the balance between lab practical classes and talks?

- 4

Q6: Is there anything you would like to have learned on the course that was not included?

More detail about CRISPR, with a focus on expressing the Cas9-gRNA system optimally.

Q7: How did you find the social activities on the course?

Mostly excellent, though still disappointed that Rob never bought us shots.

Q8: If you were in charge next year, how would you change the course?

I'd find sponsors with more interesting talks.

Q9: Would you recommend the course to someone else? Why/Why Not?

Yes. It was a great learning experience with great people.

Q10: In one or two sentences, summarise what you would tell our funders about your experience on the course.

It was a high value course packed with good into about the latest techniques in synthetic biology.

Q1: How would you describe your current position?

- Postgraduate - Masters or PhD student

Q2: Did you receive a bursary and/or travel award?

- No

Q3: How would you rate the Synthetic Genome Summer Course overall?

- 5

Q4: Are you likely to find what you learned on the Synthetic Genome Summer Course useful for your future research or industrial work, and if so how?

I anticipate that I will be able to use SCRaMbLE, the GC Prep and Benchling to help complete my lab work and thesis.

Q5: How would you rate the balance between lab practical classes and talks?

- 4

Q6: Is there anything you would like to have learned on the course that was not included?

I would have liked a more in depth tutorial on making CRISPR plasmids and primers. I appreciate that the days were already packed with activities, but I still don't feel 100% confident in these "nitty-gritty" aspects of CRISPR

Q7: How did you find the social activities on the course?

Perfect! Even though we were usually out from essentially 7:30am till midnight, the social activities (and indeed the lab work) seemed to rejuvenate and sustain us so that we could do it again every day. I've been told that I also need to mention "Shots on Rob" :D

Q8: If you were in charge next year, how would you change the course?

I would like to see a more intermediate level of lab work. Like a SGSS 2.0 where we can come back and learn the next level of SCRaMbLE, CRISPR etc. I might also try to keep the name of the course consistent. We had "summer school", "summer course" and "summer workshop". But that's just a tiny (and relatively insignificant) thing :)

Q9: Would you recommend the course to someone else? Why/Why Not?

I definitely would recommend this course purely based on how much of an amazing time I had. This was due to the fact that it was well organised and that we were looked after very, very well. The organisers went above and beyond to look after each and every one of us. Needless to say that the skills learnt, and friendships made along the way equally contributed.

Q10: In one or two sentences, summarise what you would tell our funders about your experience on the course.

Drs Tom Ellis & Ben Blount, and the team from Imperial College (Maureen, Rob & Will), put together a first class course that helped consolidate synthetic biology applications as well as forge new friendships and networks from around the world. I am truly grateful that I was selected to attend this course and if there was an opportunity for me to be involved again, I would gladly take it.

Q1: How would you describe your current position?

- Postgraduate - Masters or PhD student

Q2: Did you receive a bursary and/or travel award?

- No

Q3: How would you rate the Synthetic Genome Summer Course overall?

- 5

Q4: Are you likely to find what you learned on the Synthetic Genome Summer Course useful for your future research or industrial work, and if so how?

Maybe not really, because I have already been working with yeast for quite some time. But it might help me to find a postdoc position :)

Q5: How would you rate the balance between lab practical classes and talks?

- 4

Q6: Is there anything you would like to have learned on the course that was not included?

how to actually design CRISPR- and Golden Gate experiments

Q7: How did you find the social activities on the course?

Great! But some time off for sightseeing would have been nice.

Q8: If you were in charge next year, how would you change the course?

I would talk about the experiments in more detail, because sometimes I felt like I just put stuff together without really having background knowledge

Q9: Would you recommend the course to someone else? Why/Why Not?

YES! Because it was fun! Lots of information about Sc 2.0 and working with yeast is always cool

Q10: In one or two sentences, summarise what you would tell our funders about your experience on the course.

Respondent skipped this question

Q1: How would you describe your current position?

- Industry Researcher

Q2: Did you receive a bursary and/or travel award?

- No

Q3: How would you rate the Synthetic Genome Summer Course overall?

- 4

Q4: Are you likely to find what you learned on the Synthetic Genome Summer Course useful for your future research or industrial work, and if so how?

Yes we would like to trial the yeast golden gate kit and the synthetic yeast for easier cell line generation.

Q5: How would you rate the balance between lab practical classes and talks?

- 3

Q6: Is there anything you would like to have learned on the course that was not included?

I think it would have been good to have a bit more in depth Q&A sessions on the key topics e.g. CRISPR - maybe break out sessions?

Q7: How did you find the social activities on the course?

Brilliant! I think there was something different every evening and you got plenty of opportunity to network during the evenings

Q8: If you were in charge next year, how would you change the course?

I think for the really experienced lab workers the lab work could be a bit more challenging - maybe dividing people into two groups doing different experiments? And maybe smaller break out session discussing the more technical side of the techniques used for the lab work

Q9: Would you recommend the course to someone else? Why/Why Not?

Yes definitely - it was a great opportunity to network with not only 'yeast people', but also see science from other perspectives too. Also it was great to have a chat with the persons working on the synthetic yeast chromosome

Q10: In one or two sentences, summarise what you would tell our funders about your experience on the course.

It was just great both to see and hear all about the amazing work being done on the synthetic yeast project. It was also brilliant to be able to do some practical work so all in all the course was a great mix of talks, lab work and networking!

Q1: How would you describe your current position?

- Academic - Postdoc/Fellow/PI

Q2: Did you receive a bursary and/or travel award?

- No

Q3: How would you rate the Synthetic Genome Summer Course overall?

- 5

Q4: Are you likely to find what you learned on the Synthetic Genome Summer Course useful for your future research or industrial work, and if so how?

Yes. By learning how to people in the SC2.0 community designed and built the synthetic yeast genome will give better ideas for projects involving the synthesis of other genomes.

Q5: How would you rate the balance between lab practical classes and talks?

- 3

Q6: Is there anything you would like to have learned on the course that was not included?

Hard to say. Probably not as the planning was already dense enough

Q7: How did you find the social activities on the course?

Great! I would not have expected something better.

Q8: If you were in charge next year, how would you change the course?

1) maybe ask each participant to have one/2 slides on their projects for a 2-minute presentation. This could help to have a better idea on what each person is doing or interested in. 2) have a session that allow people to discuss in small groups on how to apply what they have learned in the course in their respective projects. Interesting discussions/collaborations may start after this.

Q9: Would you recommend the course to someone else? Why/Why Not?

Probably yes. I would rather recommend the course to undergrad or phD student starting project in the field. Lab classes were adequate to the broad audience but people with more lab experience may not find these classes really useful.

Q10: In one or two sentences, summarise what you would tell our funders about your experience on the course.

Amazing and unique opportunity to learn on synthetic genomes in a scientifically, socially and geographically wonderful environment.

Q1: How would you describe your current position?

- Postgraduate - Masters or PhD student

Q2: Did you receive a bursary and/or travel award?

- No

Q3: How would you rate the Synthetic Genome Summer Course overall?

- 5

Q4: Are you likely to find what you learned on the Synthetic Genome Summer Course useful for your future research or industrial work, and if so how?

Yes. It broadened my general knowledge about S. cerevisiae as well as teached me some very useful tools.

Q5: How would you rate the balance between lab practical classes and talks?

- 3

Q6: Is there anything you would like to have learned on the course that was not included?

No. The scope was quite broad for such a limited amount of time.

Q7: How did you find the social activities on the course?

Wonderful. Everything was taken care for and it exceeded my expectations by far. Also both the organizers and the people attending were very nice.

Q8: If you were in charge next year, how would you change the course?

Include a resting day between the course and the conference or at least an afternoon of to see the city.

Q9: Would you recommend the course to someone else? Why/Why Not?

Definitely yes. It's a nice way to learn more from a lot of different people with different specialties. Furthermore, it's a convenient way to network and make new friends.

Q10: In one or two sentences, summarise what you would tell our funders about your experience on the course.

Tom, Ben, Maureen, Rob and Will did a great job in teaching 30 people from a whole range of specialties and countries how some of the future tools for S. cerevisiae work, familiarize them with the tools and give them a chance to both meet relevant industrial companies as well as fellow scientists from many different labs.

Q1: How would you describe your current position?

- Academic - Postdoc/Fellow/PI

Q2: Did you receive a bursary and/or travel award?

- No

Q3: How would you rate the Synthetic Genome Summer Course overall?

- 5

Q4: Are you likely to find what you learned on the Synthetic Genome Summer Course useful for your future research or industrial work, and if so how?

mostly being inspired, but not really apply actual knowledge

Q5: How would you rate the balance between lab practical classes and talks?

- 4

Q6: Is there anything you would like to have learned on the course that was not included?

not really

Q7: How did you find the social activities on the course?

excellent

Q8: If you were in charge next year, how would you change the course?

more lab work

Q9: Would you recommend the course to someone else? Why/Why Not?

sure, great experience

Q10: In one or two sentences, summarise what you would tell our funders about your experience on the course.

Very well organized summer school-introduction to cutting-edge techniques. I had a great and very constructive time.

Q1: How would you describe your current position?

- Academic - Postdoc/Fellow/PI

Q2: Did you receive a bursary and/or travel award?

- No

Q3: How would you rate the Synthetic Genome Summer Course overall?

- 5

Q4: Are you likely to find what you learned on the Synthetic Genome Summer Course useful for your future research or industrial work, and if so how?

It's made me more interested in the field and even though I may never lead/conceive of a project in it, I can imagine collaborating with others who are, and will have a better understanding of the terms/techniques involved.

Q5: How would you rate the balance between lab practical classes and talks?

- 3

Q6: Is there anything you would like to have learned on the course that was not included?

More hands on examples (with a comp. lab session) of actually designing crispr constructs rather than just a quick demo

Q7: How did you find the social activities on the course?

Very well organised! Especially the games night.

Q8: If you were in charge next year, how would you change the course?

I'd space out the industry talks more so that there could be a mix of things and possibly a routine each day (acad talks 11-1 say, lab work when needed, industry talks in the afternoon possibly?). Also - invite the academic presenters and industry speakers to the social events (more time to chat about things offline).

Q9: Would you recommend the course to someone else? Why/Why Not?

Absolutely. Learnt a lot in very little time, alongside and from very nice people!

Q10: In one or two sentences, summarise what you would tell our funders about your experience on the course.

This was easily the best summer school I have ever been to. I learnt more than I thought possible about synthetic genomes in general and the Yeast 2.0 project in particular.

Q1: How would you describe your current position?

- Postgraduate - Masters or PhD student

Q2: Did you receive a bursary and/or travel award?

- No

Q3: How would you rate the Synthetic Genome Summer Course overall?

- 5

Q4: Are you likely to find what you learned on the Synthetic Genome Summer Course useful for your future research or industrial work, and if so how?

As I work on pathway engineering in yeast it was very useful for me.

Q5: How would you rate the balance between lab practical classes and talks?

- 3

Q6: Is there anything you would like to have learned on the course that was not included?

Maybe the computer section with benchling could be a bit longer to learn more about it.

Q7: How did you find the social activities on the course?

I think it was great. Every dinner was really delicious. Maybe an additional city tour would be perfect.

Q8: If you were in charge next year, how would you change the course?

I think the course is organized really good and Ben, Maureen and Rob did a great job! Nothing to change!

Q9: Would you recommend the course to someone else? Why/Why Not?

Yes of course! Because I really learned a lot and the organization of everything was great.

Q10: In one or two sentences, summarise what you would tell our funders about your experience on the course.

I learned a lot about current yeast techniques and could make contact with many interesting scientists from my field.

Q1: How would you describe your current position?

- Academic - Postdoc/Fellow/PI

Q2: Did you receive a bursary and/or travel award?

- No

Q3: How would you rate the Synthetic Genome Summer Course overall?

- 5

Q4: Are you likely to find what you learned on the Synthetic Genome Summer Course useful for your future research or industrial work, and if so how?

Yes, very useful for my ongoing academic research

Q5: How would you rate the balance between lab practical classes and talks?

- 3

Q6: Is there anything you would like to have learned on the course that was not included?

No

Q7: How did you find the social activities on the course?

Excellent and very well organised

Q8: If you were in charge next year, how would you change the course?

Going seven days from Sunday to Saturday was pretty exhausting.

Q9: Would you recommend the course to someone else? Why/Why Not?

Yes, if they were interested in synthetic yeast

Q10: In one or two sentences, summarise what you would tell our funders about your experience on the course.

Very well organised by an extremely helpful and well-informed team. A great experience.
